# Knowledge of Malaria and Its Association with Malaria-Related Behaviors—Results from the Malaria Indicator Survey, Ethiopia, 2007

**DOI:** 10.1371/journal.pone.0011692

**Published:** 2010-07-21

**Authors:** Jimee Hwang, Patricia M. Graves, Daddi Jima, Richard Reithinger, S. Patrick Kachur, and the Ethiopia MIS Working Group

**Affiliations:** 1 U.S. Centers for Disease Control and Prevention, Atlanta, Georgia, United States of America; 2 Global Health Group, UCSF Global Health Sciences, San Francisco, California, United States of America; 3 The Carter Center, Atlanta, Georgia, United States of America; 4 Ethiopian Health and Nutrition Research Institute, Addis Ababa, Ethiopia; 5 U.S. Agency for International Development, Addis Ababa, Ethiopia; Columbia University, United States of America

## Abstract

**Background:**

In 2005, the Ministry of Health in Ethiopia launched a major effort to distribute over 20 million long-lasting insecticidal nets, provide universal access to artemisinin-based combination therapy (ACTs), and train 30,000 village-based health extension workers.

**Methods and Findings:**

A cross-sectional, nationally representative Malaria Indicator Survey was conducted during the malaria transmission season in 2007. Multivariate logistic regression analyses were performed to assess the effect of women's malaria knowledge on household ITN ownership and women's ITN use. In addition, we investigated the effect of mothers' malaria knowledge on their children under 5 years of age's (U5) ITN use and their access to fever treatment on behalf of their child U5. Malaria knowledge was based on a composite index about the causes, symptoms, danger signs and prevention of malaria. Approximately 67% of women (n = 5,949) and mothers of children U5 (n = 3,447) reported some knowledge of malaria. Women's knowledge of malaria was significantly associated with household ITN ownership (adjusted Odds Ratio [aOR] = 2.1; 95% confidence interval [CI] 1.6–2.7) and with increased ITN use for themselves (aOR = 1.8; 95% CI 1.3–2.5). Knowledge of malaria amongst mothers of children U5 was associated with ITN use for their children U5 (aOR = 1.6; 95% CI 1.1–2.4), but not significantly associated with their children U5 seeking care for a fever. School attendance was a significant factor in women's ITN use (aOR = 2.0; 95% CI 1.1–3.9), their children U5′s ITN use (aOR = 4.4; 95% CI 1.6–12.1), and their children U5 having sought treatment for a fever (aOR = 6.5; 95% CI 1.9–22.9).

**Conclusions:**

Along with mass free distribution of ITNs and universal access to ACTs, delivery of targeted malaria educational information to women could improve ITN ownership and use. Efforts to control malaria could be influenced by progress towards broader goals of improving access to education, especially for women.

## Introduction

Malaria is a leading cause of morbidity and mortality in Ethiopia [1]. In 2007–2008, malaria was the top cause of outpatient visits and admissions with 12% of all visits and 10% of admissions [2]. Malaria transmission intensity varies throughout Ethiopia and is mainly determined by its diverse eco-climatic conditions. Climatic factors such as temperature, rainfall, and humidity vary greatly, partly as a function of altitude. In general, areas below 2,000 m elevation are considered at risk for malaria and are targeted to receive malaria interventions. This encompasses approximately 75% of the country's landmass and 68% of the population [3].

Ethiopia was one of the first countries to embrace the concepts of scale up for impact (SUFI). With its 2006–2010 National 5-Year Strategic Plan for Malaria Prevention and Control, Ethiopia committed to an unprecedented 100% access to effective and affordable antimalarial treatment and 100% coverage with, on average, two insecticide-treated nets (ITNs) per household by the end of 2010 [4]. Achieving this SUFI goal involved universal access to artemether-lumefantrine, the first-line drug for *Plasmodium falciparum*, distributing over 20 million long lasting insecticidal nets (LLINs), and training over 30,000 health extension workers in malaria diagnosis and treatment at the community level. To evaluate this massive scale up, Ethiopia undertook a Malaria Indicator Survey (MIS) during the country's main transmission season in 2007. The MIS showed that since 2005 Ethiopia increased household ITN ownership of at least one ITN per household from 3% [5] to 53% by the end of 2007 [6], [7]. ITN use by children under 5 years of age (U5) also improved tremendously from 2% [5] to 33% [6], [7]. Among children U5, 22% reported a fever in the two weeks prior to the survey, but of those with fever, only 10% took an antimalarial drug [6], [7]. Although huge progress has been made in scaling up key malaria interventions, Ethiopia falls short of reaching either Roll Back Malaria (RBM)'s target goal of 80% coverage for key interventions [8] or Ethiopia's even more ambitious goal of 100% household ITN ownership and 100% access to diagnosis and treatment for fevers in children U5 within 24 hours [3].

Methods to increase ITN ownership and use through private sector or subsidized schemes have largely failed to address socioeconomic inequities, with wealthier population groups reporting higher ownership and use of ITNs [9], [10], [11]. In several settings including Ethiopia, these inequities were only resolved after free mass distribution [6], [11], [12].To complement the intervention scale up, Ethiopia is now implementing broad-reaching policy changes and universal delivery of preventive and curative services. However, to achieve impact on malaria morbidity and mortality, communities will have to access and use these services. A review of household surveys from several sub-Saharan African countries noted that the proportion of children U5 who slept under a net the night before the survey was considerably lower than the proportion of households that possess a net [13]. In western Kenya following distribution of ITNs and complementary educational activities to emphasize ITN use, approximately 30%–50% of nets were still unused [14], [15].

Previous studies have identified factors for improving malaria related decisions and health behaviors. Differences in ITN ownership have been associated with malaria knowledge, women's and head of household's education, head of household's occupation, marital status, household size, household wealth, living in rural areas, and expenditure on other malaria prevention products and practices [10], [16], [17]. Similarly, malaria knowledge, women's age, higher educational attainment, household wealth, travel time to nearest market center, seasonality and weather have been reported to be important determinants of ITN use [10], [14], [16], [18], [19], [20]. Other studies have shown that greater malaria knowledge, education and wealth are not consistent determinants of net use [14], [21], [22]. Seeking health facility-based care for children U5 with fevers has been associated with multiple factors such as older age of the mother, younger age of the child, household wealth, access to health care facility, urban residence, cost, severity of illness, mother's education, head of household's education, and access to media [18], [23], [24], [25], [26], [27], [28], [29]. The relative importance of these demographic and socioeconomic factors varies among the different geographic and epidemiologic settings.

Many of these previously identified determinants of ITN ownership and use and care-seeking behavior were demonstrated prior to major scale-up of malaria interventions. Determining additional factors, especially modifiable factors, that drive ITN use once the important hurdle of ITN possession has been overcome and fever treatment seeking once ACTs are made freely available, will be vital to improving the impact of these malaria interventions. Using data from the MIS, we wanted to examine how -in the Ethiopian context of SUFI- malaria knowledge along with other modifiable factors is associated with malaria-related health behaviors. The health-related behaviors investigated in this study were *(i)* household ownership of at least one ITN; *(ii)* woman's use of ITN; *(iii)* ITN use by child U5; and *(iv)* fever treatment seeking for child U5.

## Methods

### Study area and survey

The MIS, a cross-sectional, nationally representative survey following RBM Monitoring and Evaluation Reference Group (MERG) guidelines, was conducted in the main malaria transmission season of 2007, from October–December. The principal objectives of the survey were to measure the coverage and use of malaria interventions, including ITNs, indoor residual spraying (IRS) and antimalarial drugs, as well as estimate the prevalence of fever, malaria parasitemia, and anemia. All enumeration areas (EAs) in the country with a median altitude below 2,500 m were stratified into below 1,500 m and between 1,500 m and 2,500 m altitude categories. EAs located between 1,500 m and 2,500 m were further stratified by urban and rural categories.

The details of the survey have been described previously in full [6], [7]. In brief, 319 EAs were selected based on probability proportional to size and then 25 households per each EA randomly selected. Personal digital assistants (PDAs) were used for the random sampling of households and recording of household interviews, malaria rapid diagnostic test (RDT) and hemoglobin results[30].

The questionnaires included a household and a women's sections, which were adapted to the local context from the MIS questionnaires developed by RBM MERG [5], [6]. The household questionnaire was administered to the head of the household to assess socio-economic characteristics and household coverage of malaria interventions. If women between the ages of 15–49 years were identified, the women's questionnaire which included their background characteristics, reproduction history, knowledge, attitude, and practices (KAP) around malaria, and fever history in children U5 was completed. Although the standard RBM MERG women's questionnaire does not include an assessment of knowledge, several countries have adapted the questionnaire to include this section [31], [32]. Women's knowledge of malaria was assessed by asking unprompted questions about the cause of malaria, signs and symptoms of malaria infection, signs and symptoms to suggest severe malaria, and ways to prevent malaria. The differences in answering unprompted questions (recall) compared to prompted (recognition) are well recognized and in general, response levels for prompted questions are higher, but less accurate [33], [34], [35]. Unprompted questions were used in this setting to measure recall as a more stringent and accurate measure of knowledge [33]. A woman's exposure to malaria educational information and, specifically, the setting and type of messaging heard were also assessed.

### Data analysis

PDA data were downloaded into Microsoft Access 2003 (Microsoft, Redmond, WA, USA) and imported into SAS 9.2 (SAS Institute Inc., Cary, NC, USA) for data management and statistical analyses. ITNs were defined as any LLIN or a conventional net treated with insecticides in the past 12 months. ITN use was defined as reported sleeping under an ITN the night prior to the survey. Household wealth quintiles were derived from a principal components analysis of ownership of durable assets, housing materials (e.g. roof, wall, and floor), and type of access to water and sanitation. The first principal component with its associated variance was used to rank the households.

The knowledge section included one question each assessing the cause, symptoms, danger signs, and preventive measures of malaria. Each unprompted question had a differing number of potential correct answers and respondents were allowed to give more than one answer. Varying methods of calculating composite scores and categorizing them from quantitative knowledge data have increasingly been used [19], [33],[36],[37]. In the present study, a composite malaria knowledge score was calculated for each woman where every correct answer received a single point. This score was then dichotomized to women with no correct knowledge of malaria (score = 0) and those with any correct knowledge of malaria (score ≥1). Although a ranking of malaria knowledge derived from the principal components analysis showed a Pearson's correlation coefficient of 0.9 with this composite knowledge score (data not shown), the simpler to comprehend and calculate composite knowledge score was used to summarize women's knowledge.

Survey procedures within SAS adjusted for multi-stage sampling, clustering, and weighting for selection probabilities. Households with more than one eligible woman or mothers with more than one child U5 were taken into account in the overall weighting.

Descriptive statistics were used to explore the characteristics of all women and a subset of women who were mothers of children U5. Significant differences between the two groups were tested using chi-square and t-test for ordinal and continuous data, respectively. Univariate analyses of specific areas of malaria knowledge and receiving malaria information were performed, but not included in the multivariate model due to its correlation with the overall malaria knowledge variable. Multivariate logistic regression analyses were performed to assess women's malaria knowledge as it predicted household ownership of any ITNs and her own use of an ITN the night prior to the survey, with variables eliminated from the full model in order of least significance. ITN use and fever treatment in children U5 were also explored in terms of their mother's knowledge of malaria. All analyses of ITN use were restricted to households that owned at least one ITN.

Along with the explanatory variables, the following potential two-way interaction terms were included in the full model: malaria knowledge with household wealth quintiles, malaria knowledge with women's history of school attendance, women's school attendance with household wealth quintiles, and EA category (urban vs. rural) with school attendance. For terms that suggested the presence of an interaction (p<0.10), stratified analyses to assess the role of such effect modifiers were performed in order to interpret these associations in programmatically relevant terms.Results of the interaction terms are presented as p-values because of the difficulty of interpreting the odds ratio (OR) of an interaction term [38].

### Ethics Statement

The MIS protocol received ethical clearance from the Emory University Institutional Review Board (IRB#6389), the CDC Ethical Review (IRB#990132), the Program for Appropriate Technology in Health (PATH) Ethical Committee, and the Ethiopian Science and Technology Agency. Verbal informed consent, which was approved by the above ethical boards and recorded in the PDA, was obtained from the heads of the household to participate in the household questionnaire, each eligible woman to participate in the women's questionnaire, and again from every individual prior to blood sample collection. Additional verbal assent was obtained from children 6 to 18 years of age.

## Results

### Characteristics of eligible women

Table 1 shows the comparative household and individual characteristics in the survey for all women and a subset of those women who were mothers of children U5. Data from a total of 6,657 women (5,949 households) which included 4,714 mothers of children U5 (3,447 households) were analyzed. Fewer of the latter lived in urban EAs (11.7%) compared to all women (17.2%). Mothers of children U5 also lived in larger households (5.5 mean number of household members) compared to all women (4.9 mean number of household members). Fewer (15.0%) mothers of children U5 lived in households of the highest wealth quintiles compared to all women (20.1%), while more (57.4%) mothers of children U5 lived in households that owned at least one ITN.

**Table 1:** Household and individual characteristics for all women and subset of mothers of children under 5 years of age (U5), Ethiopia, 2007.

Characteristic	All women (95% CI)	Mothers of children U5 (95% CI)	p-value
**Household Characteristics**			
Number of Households (N)	5949	3447	
Residence in an urban EA (%)	17.2 (12.9–21.4)	11.7 (8.2–15.2)	<.0001
Residence in EA with altitude 2000–2500 m (%)	32.2 (25.7–38.7)	31.4 (24.6–38.3)	0.4
Mean household size	4.9 (4.7–5.2)	5.5 (5.2–5.8)	<.0001
Wealth Quintiles			<.0001
First- Lowest	19.4 (15.6–23.1)	21.1 (16.7–25.5)	
Second	22.7 (19.3–26.1)	25.2 (21.4–29.0)	
Third	18.8 (16.2–21.3)	19.5 (16.6–22.3)	
Fourth	19.0 (16.3–21.7)	19.3 (16.1–22.5)	
Fifth- Highest	20.1 (16.2 24.1)	15.0 (11.7–18.2)	
HH with at least one ITN (%)	55.1 (49.1–61.2)	57.4 (50.9–64.0)	0.008
HH sprayed with insecticide in the past 12 months (%)	14.6 (10.4–18.8)	14.9 (10.4–19.5)	0.6
**Individual Characteristics**			
Number of Women	6657	4714	
Mean age	28.0 (27.6–28.4)	28.2 (27.7–28.6)	0.07
Attended any school (%)	23.7 (20.3–27.2)	16.9 (14.0–19.7)	<.0001
Some level of literacy (%)	15.7 (13.4–18.0)	12.5 (10.3–14.7)	<.0001
Ever heard of malaria (%)	73.8 (70.0–77.7)	74.2 (70.0–78.3)	0.3
Knew mosquitoes caused malaria (%)	34.6 (31.0–38.3)	33.6 (29.6–37.7)	0.2
Knew nets/ITN prevented malaria (%)	31.5 (27.8–35.2)	30.3 (26.2–34.5)	0.1
Knew fever as symptom of malaria (%)	43.6 (39.6–47.6)	43.1 (38.6–47.5)	0.6
Knew at least one danger sign of malaria (%)	39.7 (35.8–43.7)	38.5 (34.2–42.7)	0.03
Any correct malaria knowledge (%)	67.2 (63.2–71.1)	67.6 (63.3–71.8)	0.6
Slept under an ITN the night prior to the survey (%)	33.7 (28.7–38.6)	37.5 (31.6–43.4)	0.003

U5 =  under 5 years of age; CI = Confidence Intervals; EA = Enumeration area; HH = Household; ITN = Insecticide-treated nets.

More women in general attended any school (23.7%) and had some level of literacy (15.7%) compared to women who were mothers of children U5 (16.9%; 12.5%, respectively). Although, 5,023/6,657 (73.8%) women had heard of malaria, only 2,953 (43.6%) stated that fever was a symptom, 2,233 (34.6%) mentioned mosquitoes as the cause, 2,296 (31.5%) reported that nets or ITNs could prevent malaria, and 2,731 (39.7%) named at least one danger sign of malaria. Overall 4,573 (67.6%) women and 3,193 (67.2%) mothers of children U5 answered at least one malaria knowledge question correctly, which was not significantly different between the two groups (Table 1).

Of the 1,800 women from 1,716 households who could not answer any malaria knowledge question correctly, fewer lived in urban EAs (8.4% versus 17.2% of all women, Table 1), belonged to highest wealth quintiles (13.0% versus 20.1%), or attended any school (13.1% versus 23.7%).

### Source and type of malaria information

Of all women, only 25.4% (n = 1946) had seen or heard messages about malaria and even fewer 4.3% (n = 410) had received malaria information in their homes (Table 2). Friends and family (33.7%) and government clinics or hospitals (28.9%) were the most common sources of malaria information. Print media e.g. posters, billboards, and newspapers were cited least often. The importance of sleeping under a net or ITN (58.3%) and environmental sanitation (41.0%) were the most often heard messages.

**Table 2:** Summary of malaria information received amongst women age 15–49 years, Ethiopia, 2007.

Characteristic	N	Percent (%)	95% CI
Number of women	6657		
Seen or heard messages about malaria	1946	25.4	21.8–29.0
Source of malaria information			
Friends/Family	570	33.7	27.7–39.7
Government clinic/hospital	689	28.9	23.6–34.2
Health Extension Worker	336	15.9	11.6–20.1
On the radio	262	13.7	8.6–18.9
On television	143	9.2	4.3–14.0
Peer educators	108	6.2	3.6–8.7
Workplace	68	3.2	1.8–4.5
In print (Posters/Billboards/Newspaper)	32	2.5	1.3–3.7
Type of malaria information received			
Sleeping under net/ITN	1193	58.3	51.7–64.8
Environmental sanitation	832	41.0	35.9–46.2
Seek treatment for fever	341	21.8	16.9–26.7
Importance of spraying	154	5.8	4.0–7.5
Received malaria information at home	410	4.3	3.1–5.5
Type of malaria information received at home			
Sleeping under net/ITN	295	63.5	51.6–75.4
Environmental sanitation	171	45.2	34.5–55.8
Seek treatment for fever	38	12.0	5.9–18.1
Importance of spraying	21	3.8	0.8–6.8

CI = Confidence Intervals; ITN = Insecticide-treated nets.

### Household ownership of ITNs

Of the 5,949 households with eligible women, 3,463 (55.1%) lived in a household with at least one ITN (Table 1). Univariate analyses of specific knowledge areas showed that knowing mosquitoes as the cause (OR = 1.3 [95% confidence interval (CI) 1.0–1.7]), and nets/ITNs as a preventive measure of malaria (OR = 2.3 [95% CI 1.7–3.0]) were significantly associated with ITN ownership (Table 3). Having seen or heard malaria messages was not significantly associated with ITN ownership (OR = 1.4 [95% CI 1.0–1.9]) while receiving malaria messages at home was significant (OR = 3.1 [95% CI 1.9–5.3]).

**Table 3:** Univariate analysis of aspects of malaria knowledge and its association with specific health-related behaviors.

	Odds Ratio (95% Confidence Intervals)			
Knowledge Characteristic	Household Owns ≥1 ITN	Woman's use of ITN	Child U5′s use of ITN	Fever treatment for Child U5
Nets/ITN for prevention	2.3 (1.7–3.0)*	1.5 (1.1–1.9)*	1.5 (1.0–2.1)*	1.5 (1.0–2.3)*
Mosquitoes as cause	1.3 (1.0–1.7)*	1.2 (1.0–1.5)	1.1 (0.7–1.5)	2.3 (1.5–3.5)*
Fever as a symptom	1.9 (1.5–2.5)*	1.3 (1.0–1.6)	1.1 (0.8–1.4)	1.3 (0.9–2.0)
Any correct danger signs/symptoms	1.7 (1.3–2.2)*	1.4 (1.1–1.9)*	1.4 (1.0–2.0)*	1.0 (0.7–1.6)
Received malaria message	1.4 (1.0–1.9)	1.1 (0.9–1.4)	1.1 (0.8–1.5)	1.3 (0.8–2.0)
Received malaria message at home	3.1 (1.9–5.3)*	2.5 (1.5–4.2)*	2.3 (1.1–4.9)*	1.2 (0.6–2.4)

*p-value <0.05.

ITN = Insecticide-treated nets; U5 =  under 5 years of age.

Multivariate analysis showed that ITN ownership was significantly associated with any malaria knowledge (adjusted OR [aOR] 2.1 [95% CI 1.6–2.7]), EA altitude (aOR 0.3 [95% CI 0.2–0.5]), household sprayed with insecticide (aOR 4.6 [95% CI 2.6–8.2]), and an interaction term between EA category and school attendance (p = 0.03) (Table 4). Residence in an urban or rural EA category predicting ITN ownership depends on whether the woman attended any school.

**Table 4:** Women's knowledge of malaria as it predicts household ownership of ITNs.

Risk Factor	Total (n)	% ITN own	Multivariate*		
			aOR	95% CI	p-value
***Individual characteristics***					
Any malaria knowledge					
No	2084	41.2			
Yes	4573	61.9	2.1	1.6–2.7	<0.0001
Attended school					
No	4925	57.4			
Yes	1732	47.9	0.9	0.6–1.3	0.6
***Household characteristics***					
EA Category					
Rural	5292	57.6			
Urban	1365	43.2	0.9	0.5–1.6	0.7
EA altitude					
<2000 m	4466	67.6			
2000–2500 m	2191	28.9	0.3	0.2–0.5	<0.0001
HH sprayed with insecticide in the past 12 months					
No	5692	49.4			
Yes	891	87.7	4.6	2.6–8.2	<0.0001
***Interaction terms***					
EA Category*Attended any school					0.03

*The full multivariate model included the following non-significant terms: woman's age in 5 year increments, woman with child under 5 years of age, household size, wealth index, and interaction terms (any malaria knowledge and school attendance, any malaria knowledge and wealth index, and school attendance and wealth index).

aOR =  adjusted odds ratio; CI = Confidence Intervals; EA = Enumeration area; HH = Household; ITN = Insecticide-treated nets.

### Women's use of ITNs

Of the 6,657 women surveyed, 3,463 (33.7%) slept under an ITN the night prior to the survey (Table 1). Of the women who had slept under an ITN the night prior to the survey, 49.7% (1,084/2,359) slept under the same ITN as their child U5. Restricting all analyses to households with at least one ITN, knowing nets/ITNs could prevent malaria (OR = 1.5 [95% CI 1.1–1.9]) as well as receiving malaria messages at home (OR = 2.5 [95% CI 1.5–4.2]) were significantly associated with women's ITN use (Table 3).

The multivariate analysis which was also restricted to households with at least one ITN showed that women's use of ITNs were significantly associated with malaria knowledge (aOR 1.8 [95% CI 1.3–2.5]), having a child U5 (aOR 1.9 [95% CI 1.5–2.4]), woman's school attendance (aOR 2.0 [95% CI 1.1–3.9]), residence in an urban EA category (aOR 1.7 [95% CI 1.1–2.5]), household size (aOR 0.8 [95% CI 0.8–0.9]), household sprayed with insecticide in the past 12 months (aOR 1.8 [95% CI 1.3–2.5]), number of ITNs in the household (aOR 1.7 [95% CI 1.4–2.0]), and interaction terms between correct malaria knowledge and school attendance (p = 0.04) and between EA category and school attendance (p = 0.01) (Table 5). In assessing the interaction terms (Table 5), both malaria knowledge and EA category were significant predictors of woman's ITN use only if the woman had not attended any school.

**Table 5:** Women's knowledge of malaria as it predicts their ITN use in households that own at least one ITN.

Risk Factor	Total (n)	% ITN use	Multivariate*		
			aOR	95% CI	p-value
***Individual characteristics***					
Any malaria knowledge					
No	932	50.9			
Yes	2970	64.4	1.8	1.3–2.5	0.0004
Attended school					
No	2954	61.2			
Yes	948	60.6	2.0	1.1–3.9	0.03
Has a child U5					
No	1803	54.6			
Yes	2099	65.4	1.9	1.5–2.4	<0.0001
***Household characteristics***					
EA Category					
Rural	717	60.5			
Urban	3185	64.3	1.7	1.1–2.5	0.009
HH sprayed in the past 12 months					
No	3092	58.0			
Yes	759	72.0	1.8	1.3–2.5	0.001
Increasing HH size	3902		0.8	0.8–0.9	<0.0001
Increasing number of ITNs in the HH	3902		1.7	1.4–2.0	<0.0001
***Interaction terms***					
Any malaria knowledge*Attended any school					0.04
EA Category*Attended any school					0.01

*The full multivariate model included the following non-significant terms: woman's age in 5 year increments, wealth index, EA altitude, and interaction terms (any malaria knowledge and wealth index, and school attendance and wealth index).

aOR =  adjusted odds ratio; CI = Confidence Intervals; EA = Enumeration area; HH = Household; ITN = Insecticide-treated nets; U5 =  under 5 years of age.

### Child's use of ITNs

1,876/5,225 (33.1%) of children U5 slept under an ITN the night prior to the survey [6], [7]. Similar to woman's use of ITNs, knowing nets could prevent malaria (OR = 1.5 [95% CI 1.0–2.1]) as well as receiving malaria messages at home (OR = 2.3 [95% CI 1.1–4.9]) were significantly associated with her child's ITN use (Table 3).

Restricting the multivariate analysis to households with at least one ITN, it showed that ITN use by children U5 was associated with their mother's malaria knowledge (aOR = 1.6; 95% CI 1.1–2.4), their mother having attended school (aOR = 4.4; 95% CI 1.6–12.1), living in an urban EA category (aOR = 1.8; 95% CI 1.1–2.8), living in a household sprayed with insecticide in the past 12 months (aOR = 1.5; 95% CI 1.1–2.2), increasing number of household ITNs (aOR = 1.6; 95% CI 1.3–2.1), and decreasing household size (aOR = 0.9; 95% CI 0.8–0.9) (Table 6). The restricted model also included a significant interaction term between any correct malaria knowledge and the mother having attended school (p = 0.04). Malaria knowledge predicting a child U5′s ITN use depends on whether his/her mother attended school. If the mother attended school, malaria knowledge was not a significant factor of U5 child's ITN use (p = 0.8); however, if the mother did not attend school, any correct knowledge of malaria was a significant factor associated with the child U5′s ITN use (p = 0.009).

**Table 6:** Knowledge of malaria amongst mothers of children U5 as it predicts ITN use for their children U5 in households that own at least one ITN.

Risk Factor	Total (n)	% ITN use	Multivariate*		
			aOR	95% CI	p-value
***Individual characteristics***					
Any malaria knowledge					
No	642	52.3			
Yes	2185	63.2	1.6	1.1–2.4	0.009
Attended school					
No	2399	58.6			
Yes	428	72.3	4.4	1.6–12.1	0.005
***Household characteristics***					
EA Category					
Rural	2721	58.1			
Urban	365	75.3	1.8	1.1–2.8	0.01
HH sprayed with insecticide in the past 12 months					
No	2432	57.5			
Yes	628	68.0	1.5	1.1–2.2	0.03
Increasing HH size	3086		1.6	1.3–2.1	0.0005
Increasing number of ITNs in the HH	3086		1.6	1.3–2.1	<0.0001
***Interaction terms***					
Any correct malaria knowledge*Attended any school					0.04

*The full multivariate model included the following non-significant terms: woman's age in 5 year increments, child living with mother, age of child, sex of child, wealth index, EA altitude, and interaction terms (EA category and school attendance, any malaria knowledge and wealth index, and school attendance and wealth index).

aOR =  adjusted odds ratio; CI = Confidence Intervals; EA = Enumeration area; HH = Household; ITNs = Insecticide-treated nets; U5 =  under 5 years of age.

### Fever treatment seeking behavior

Of the 22.3% (1,034/4,384) of children U5 with fever in the two weeks prior to the survey [6], [7], only 342/1,009 (36.5%) sought any type of treatment, which included public and private sector health facilities or hospitals, health extension workers, pharmacies, shops, and traditional healers. Knowing nets could prevent malaria (OR = 1.5 [95% CI 1.0–2.3]) and mosquitoes as the cause (OR = 2.3 [95% CI 1.5–3.5]) were significantly associated with seeking treatment, but knowing clinical features such as fever as a symptom or any danger signs were not significant.

In multivariate analysis, a mother of a child U5 having any correct malaria knowledge (aOR = 0.9; 95% CI 0.3–2.2) was not significantly associated with the child having sought treatment for a fever episode. However, increased treatment seeking was significantly associated with his/her mother having attended school (aOR = 6.5; 95% CI 1.9–22.9) and residence in an urban EA category (aOR = 4.0; 95% CI 1.9–8.3), while decreased treatment seeking was significantly associated with residence in an EA with altitude 2000–2500 m (aOR = 0.5; 95% CI 0.3–0.8) and the household owning at least one ITN (aOR = 0.5; 95% CI 0.3–0.9) (Table 7). The restricted model also included significant interaction terms between malaria knowledge and wealth index (p = 0.04) and between malaria knowledge and school attendance (p = 0.05). Again, malaria knowledge was a significant factor in predicting fever treatment seeking only amongst mothers who did not attend school. Albeit non-significant, the direction of association between malaria knowledge and fever treatment varied depending on the household wealth quintile.

**Table 7:** Knowledge of malaria amongst mothers of children U5 as it predicts access to fever treatment for their children U5 with reported fever in the past two weeks.

Risk Factor	Total (n)	% seek	Multivariate*		
			aOR	95% CI	p-value
***Individual characteristics***					
Any malaria knowledge					
No	216	29.6			
Yes	788	38.6	0.9	0.3–2.2	0.8
Attended school					
No	823	32.5			
Yes	181	54.2	6.5	1.9–22.9	0.003
Sex of Child					
Male	522	31.4			
Female	487	42.0			
***Household characteristics***					
EA Category					
Rural	904	34.0			
Urban	105	67.6	4.0	1.9–8.3	0.0002
EA Altitude					
<2000 m	726	38.4			
2000–2500 m	283	31.3	0.5	0.3–0.8	.007
HH owns at least one ITN					
No	318	41.5			
Yes	691	33.3	0.5	0.3–0.9	0.01
HH Wealth Quintiles					
First- Lowest	199	34.4	5.2	1.2–22.1	0.05
Second	272	28.3	3.0	0.8–11.2	0.6
Third	178	37.8	3.1	1.0–9.9	0.4
Fourth	224	39.8	2.3	1.0–5.2	0.7
Fifth- Highest	136	51.8			
***Interaction terms***					
Any malaria knowledge*Wealth index					0.04
Any malaria knowledge*Attended any school					0.05

*The full multivariate model included the following non-significant terms: woman's age in 5 year increments, age of child, household sprayed with insecticide in the past 12 months, and interaction terms (EA category and school attendance, and school attendance and wealth index).

aOR =  adjusted odds ratio; CI = Confidence Intervals; EA = Enumeration area; HH = Household; ITN = Insecticide-treated nets; U5 =  under 5 years of age.

## Discussion

Although a majority of women had heard of malaria in this survey, only a small proportion knew the cause, signs or symptoms, and preventive measures. We show that women belonging to the poorest wealth quintile, with no formal education, and living in rural EAs had lower levels of malaria knowledge [6]. These findings are similar to previous smaller studies in Ethiopia, which noted limited malaria knowledge amongst women [39], [40] and strong socioeconomic disparity with malaria knowledge (e.g. 96% of respondents in the highest SES quintile had heard of nets versus to 35% in the lowest quintile) [39]. These findings contrast with those reported in the Zambia MIS, where almost 100% of the women had heard of malaria, and knowledge disparities amongst wealth quintiles and education levels were less striking [31].

Women most often received their malaria information from government clinics/hospitals and from friends and family, and least often from print media. This is consistent with the very low rates of literacy reported in Ethiopia. Targeting information, education, communication/behavior change communication (IEC/BCC) efforts to women attending government clinics and hospitals could be an effective way to reach women and change their (and their household's) behavior at a time of high perceived susceptibility [41]. With the high illiteracy rates and lack of formal education, less emphasis should be placed on print media as a delivery mechanism.

Further attention is also needed on the content and types of malaria messages delivered. Less than half of the women and mothers knew fever as a symptom of malaria, or knew any danger signs of malaria. Noting the limited emphasis on fever treatment seeking in IEC activities, it is not surprising that rates of treatment seeking are unacceptably low. Increasing the quantity and improving the quality of this messaging could have a large impact on children receiving effective ACTs. Furthermore, receiving messages at home was shown to be significantly associated with improved ITN ownership and use. The 30,000 community-level health extension workers in Ethiopia represent a great opportunity to deliver targeted health messages in people's homes.

We show that ITN ownership was associated with personal or household factors, including women's malaria knowledge, residence in an EA with an altitude below 2000 m and whether the household had received other malaria interventions such as IRS. Although significant malaria transmission and net ownership still occur above 2000 m [42], malaria risk is higher below 2000 m and the increased knowledge may reflect targeting of net distribution and IEC to these areas. Malaria knowledge was associated with increased ownership of ITNs, but we cannot determine in this cross-sectional study whether this is because women had higher knowledge from exposure to more malaria interventions including ITN distribution and IRS campaigns, or because women with greater knowledge were more likely to seek out and accept malaria interventions. This important issue should be further explored through qualitative studies in the future.

The first step and most important factor driving ITN use is ITN ownership. Restricting the analysis to households that own at least one ITN, correct women's knowledge of malaria was associated with both increased ITN use for themselves and their children U5. In such households, increasing number of ITNs was associated with both women's personal use of an ITN and use by their children U5. The ratio of increasing ITNs to household size was explored across 15 standardized national surveys in Africa and generally found to be a significant factor for children U5′s ITN use [22]. A cluster randomized trial planning to evaluate the effect of training on LLIN use after LLINs were distributed in south-west Ethiopia, noted higher use with good malaria knowledge and female heads of household at baseline in some villages [43]. Improving women's malaria knowledge and increasing the number of ITNs in the household, both modifiable factors, could drive ITN use for women and children U5 once the important initial hurdle of ITN possession has been overcome.

Although recent studies, both population- and health facility-based, have suggested tremendous reductions in malaria morbidity in Ethiopia [44], [45], our survey highlighted the unacceptably low proportion of children U5 accessing any treatment for fever, or let alone prompt and effective treatment [6], [7]. Malaria knowledge was not independently associated with seeking care for fever, except for a subset of women with no prior education. For mothers of children U5, the major significant factors in determining care seeking behavior were having attended school, living in an urban area with likely improved access to care, and belonging to the highest wealth quintiles. Those living at altitudes below 2000 m and in households without an ITN were more likely to seek care, which could be attributed to varying perceptions of risk. Studies conducted in central rural Ethiopia prior to universal access to ACTs showed that only 13% [46] and 28% [47] of children U5 received any form of treatment within 24 hours. Early treatment seeking was more frequently reported by those who accessed home treatment and community health workers [47]. The recent deployment of over 30,000 health extension workers should result in large improvements in access to and use of community-based prompt and effective treatment.

Overall, women and more specifically mothers of children U5 reported very low levels of school attendance and literacy. Although women without formal schooling were receptive to malaria messages and were able to access the public health intervention, school attendance was an independent factor associated with women sleeping under an ITN, children U5 sleeping under an ITN, and fever treatment seeking. A previous study in rural Ethiopia found that literacy was a significant factor in women believing that malaria was preventable [40]. Furthermore, women's education often emerges as a key element in a strategy to improve overall child health. Formal education improves child health through directly teaching health knowledge to future mothers [48]. This is consistent with our study that malaria knowledge independent of school attendance appeared to improve malaria health-related behaviors. However, broader goals of economic development and improving access to education, especially for women remain a significant and modifiable social determinant of health-related behavior and can only aid the malaria control efforts.

Although knowledge is one aspect of a complex interplay of factors, it is an important prerequisite for instigating behavior change and could likely inform attitudes about malaria health-related behaviors. With the global efforts to achieve universal coverage by 2010 [49], programs should not only focus on delivery of goods, but packaging that with effective IEC/BCC messages. Lastly, a strategy based on a girl- or woman-centered approach, which is advocated by the Lantos-Hyde U.S. Government Malaria Strategy 2009–2014 [50], can be an effective way of increasing the emphasis on girl's education and delivering health messages to this receptive group.
